# Systemic Management of Pandemic Risks in Dental Practice: A Consolidated Framework for COVID-19 Control in Dentistry

**DOI:** 10.3389/fmed.2021.644515

**Published:** 2021-02-24

**Authors:** Habib Benzian, Eugenio Beltrán-Aguilar, Richard Niederman

**Affiliations:** ^1^Department Epidemiology and Health Promotion, World Health Organization Collaborating Center Quality Improvement and Evidence-Based Dentistry, College of Dentistry, New York University, New York, NY, United States; ^2^Global Health Center, Geneva Graduate Institute for Policy Studies, Geneva, Switzerland; ^3^Department Epidemiology and Health Promotion, College of Dentistry, New York University, New York, NY, United States

**Keywords:** infection control, workplace safety and health, hierarchy of risk control, airborne transmission of pathogens, dental procedure, systems thinking, dentistry, practice management dental

## Abstract

Dental teams and their workplaces are among the most exposed to airborne and bloodborne infectious agents, and therefore at the forefront of pandemic-related changes to how dental care is organized and provided to patients. The increasing complexity of guidelines makes is challenging for clinicians to navigate the multitude of COVID-19 guidelines issued by different agencies. A comparative analysis of guidance issued for managing COVID-19 in dental settings leading U.S. agencies was conducted, including documents of the Occupational Safety and Health Administration (OSHA), an agency of the U.S. Secretary of Labor, and of the U.S. Centers for Disease Prevention and Control (CDC), an agency of the U.S. Secretary of Health and Human Services. Details of infection control and other risk mitigation measures were reviewed for consistency, overlaps and similarities, then clustered according to thematic areas covering all domains of managing a dental healthcare setting. The analysis revealed five distinct areas of pandemic control, comprising (1) planning and protocols, (2) patient screening, (3) preparation of facilities, (4) PPE and infection control, and (5) procedures and aerosol control; thereby covering systematically all aspects requiring adaptation in a pandemic context. The “Pandemic-5 Framework for COVID-19 Control in Dentistry” provides an opportunity to simplify comprehensive decision-making from a clinical practitioner perspective. The framework supports a comprehensive systems-driven approach by using dental clinics as a setting to integrate pandemic clinical responses with the implementation of appropriate infection control protocols. Traditionally these two aspects are addressed independently from each other in separate concepts.

## Safety of Dental Care Under COVID-19

The COVID-19 pandemic has pushed safety precautions and infection control to the limelight, forcing the entire healthcare sector to review protocols and practices to ensure continued safety of care for patients and healthcare workers in an evolving context. Recognizing, understanding and managing the risks of emerging, previously unknown infections, while continuing to provide care, are complex processes. They require a systematic and systemic adaptation of multiple interlinked aspects, such as population protection and risk containment measures, healthcare service practices including infection control; and workplace health and safety for providers and patients, including surveillance of workplace-related adverse events (1–8).

Dental teams and their clinical work places are among the most affected by exposure to airborne and bloodborne infectious agents and therefore at the forefront of pandemic-related changes how dental care is organized and provided to patients (9–12). Early reports from China highlighted the risks of droplet and aerosol transmission in dental care, which led to service limitations or shut-downs in many countries worldwide, following governmental restrictions (13–15). Subsequently, numerous national recommendations for dental services have been rapidly developed, detailing adaptations of practice management, use of personal protective equipment (PPE) and other aspects of clinical dental care (15–17). In the U.S., like in many other countries, different agencies and organizations with different scopes of work are guiding infection control, workplace health and safety, and other related matters. In addition, federal and state regulations may be requiring pandemic changes to oral healthcare services.

For dental teams managing a clinical dental workplace it becomes increasingly complex to navigate this patchwork of COVID-19 guidelines issued by different agencies, also because they address different aspects of pandemic response and often reflect the organizational remit of the issuing organization. This puts a high burden on individual practitioners and practice owners. Clinicians are expected to keep themselves updated with the latest information so that they can take responsible managerial and clinical decisions to provide the safest possible healthcare environment.

This paper presents a new and consolidated framework for managing the COVID-19 pandemic risks in dental settings using five distinct areas of control. By combining approaches of different and overlapping guideline concepts of two leading U.S. public health agencies, the framework aims at simplifying decision-making and adaptations in the dental setting to mitigate the risks of COVID-19 transmission.

## Current Frameworks Addressing Occupational Risks and Infection Control in Dental Settings

In the U.S., two agencies provide public health and workplace guidance to address COVID-19: The Occupational Safety and Health Administration (OSHA), an agency of the U.S. Secretary of Labor, and the U.S. Centers for Disease Prevention and Control (CDC), an agency of the U.S. Secretary of Health and Human Services. Both have developed recommendations throughout the course of the pandemic with repeated updates accounting for the constantly evolving context. In developing these recommendations, both organizations built on their existing frameworks for risks to occupational health and infection control in healthcare and dentistry.

OSHA's “Hierarchy of Controls” addresses workplace health and safety, including healthcare settings. The concept identifies four areas of intervention with decreasing efficiency of protection for staff: Risk elimination, engineering controls, administrative practices and PPE (18). This approach has been used to issue guidance on preparing workplaces for COVID-19, as well as develop specific recommendations for “Dentistry Workers and Employers” (19, 20). OSHA places dental health care providers in the “very high” or “high” risk category because of their exposure to aerosols during dental care. For aspects of PPE and specific infection control the guidance refers to the relevant CDC recommendations.

In 2016, the CDC defined “basic expectations for safe care” in dental settings (21). The set of standard precautions comprises hand hygiene, PPE, respiratory etiquette, safe injection practice, storage and handling of instruments, and the disinfection of instruments and practice environment. In addition, a set of transmission-based precautions cover the identification of infectious patients, contact precautions, droplet and aerosol precautions. These fundamental principles are taking a clinical service provision perspective, thereby complementing OSHA's health and safety approach. The CDC's COVID-19-related recommendations for dental settings build on these standard and transmission-based precautions, but also incorporate elements of engineering controls from OSHA's framework and reference additional guidance from the American Dental Association (22, 23).

In addition, U.S. state agencies, the American Dental Association, and several dental professional associations provided further guidance, which may or may not be aligned with the advice of OSHA or the CDC. The multitude of guideline-generating agencies places dentists and their teams in a dilemma where they need to actively search for, obtain, read and compare several sources of practice recommendations. They need to appraise the quality of information provided and the relevance for their own context in order to make appropriate decisions in the best interest of patient, staff and the wider public's safety. In the current highly dynamic pandemic context this is a challenging task.

**Figure 1:**
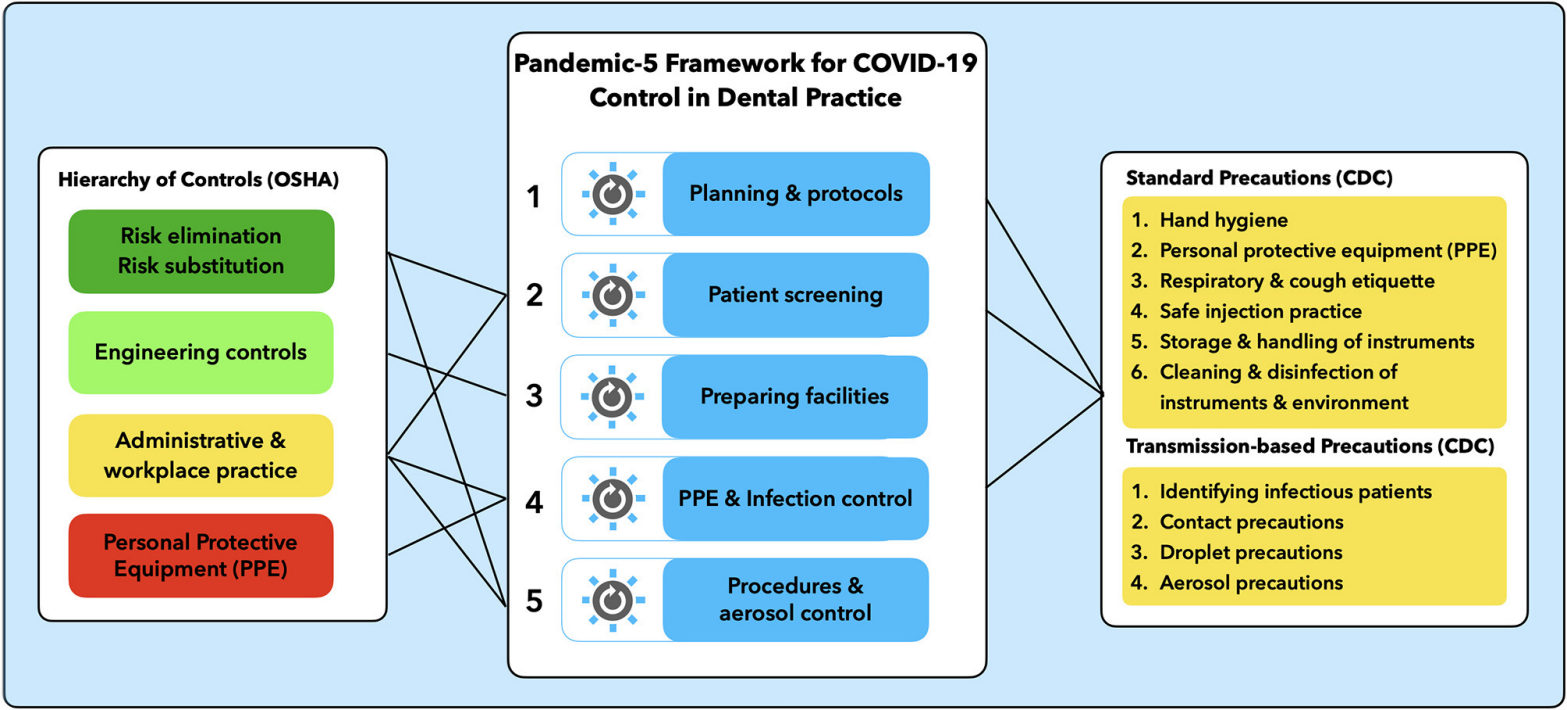
Pandemic-5 framework for COVID-19 control in dental practice.

A comparative analysis of the CDC and OSHA guidance was undertaken in order to identify areas of overlap and complementarity. Clustering of thematic areas of infection control and measures for health and safety at the workplace resulted in a consolidated framework comprising five areas of intervention. Figure 1 shows the areas of intervention of CDC's and OSHA's guidance and their respective links to the proposed new framework with five areas of control.

## A Systems-Thinking Approach to Managing Risks of COVID-19

Systems-thinking is increasingly applied to public health (24), hospital infections and infectious disease control (25, 26). Using the dental workplace as a system to analyze challenges and solutions in several interconnected and pandemic-related domains allows for a more holistic and comprehensive adaptation and response to COVID-19 in the dental practice.

The “Pandemic-5 Framework for COVID-19 Control in Dental Practice” synthesizes the elements of OSHA's “Hierarchy of Controls” and CDC's “Standard and Transmission-based Precautions” into a new, consolidated and simplified model (Figure 1).

The framework is inspired by the model for health system reform developed by Roberts et al. (27), which uses the metaphor of “control knobs” to describe core areas of intervention or change in a complex health system setting. The Pandemic-5 Framework uses the control-knob principle to systematically identify the five areas of possible interventions to manage and control the risk of COVID-19 in dental settings. The five areas and selected control interventions for each area comprise:

1. Planning and protocols
  Pandemic preparedness and response require planning and anticipation, risk assessments, thinking through different scenarios, and putting control and mitigation measures in place. Preparedness also includes communication and participation of the entire dental team and communication with patients. Staff training, rehearsing of protocols and monitoring compliance should be planned and formalized as well. Some aspects of planning and documentation may even be required as part of legal and licensing regulations. Ideally, all control measures are covered by a written plan including protocols, checklists and practical control measures.
2. Patient screening
  This area of control relates to simple assessments of the patient's health status and oral health care need. The objective is to limit or select who has access to the clinical setting. Pre-screening *via* phone or online using questionnaires and/or software are used to determine whether the patient is currently ill, has any particular risks of being infected, or is at part of a high-risk group for infection. Tele-consultations may already address the patient's problem. Through this screening process, patients with urgent problems may receive palliative care while being scheduled treatment at the dental clinic, where additional COVID-19 screening may be performed prior to providing care. Depending on the pandemic situation and infection risks, patient visits for elective procedures may be either post-poned or performed (17).
3. Preparing facilities
  The preparation of facilities comprises a range of measures to ensure physical distancing in waiting and reception areas, signage and patient flow, separation of operatories, ventilation and air filtration, enabling regular hand hygiene for patient and staff through additional disinfection dispensers. Depending on official guidance and context, additional measures to reduce aerosols and surface contamination may be taken such as spacing waiting rooms, opening windows, negative pressure rooms, or touchless doors and faucets. The area of engineering controls from OHSA's “Guidance for workplace health and safety” provides further details.
4. PPE and infection control
  Personal protective equipment is a key aspect for dental teams working in close proximity to the patient's face. A range of measures, including different types and protective levels of face masks, face shields, disposable gowns, head caps, gloves, eye protection or goggles are available and should be selected depending on the nature and length of patient contact, as well as the type of procedure performed. All of the CDC's “Standard and Transmission-based Precautions” for infection control remain in place, and additional measures for surface decontamination and operatory cleaning between patients may be recommended.
5. Procedures and aerosol control
  With the remaining uncertainty around the risk of aerosol transmission of SARS-CoV-2, dental procedures must be selected and delivered carefully. Clinicians have the choice between aerosol-generating procedures involving water-air cooled rotary instruments, ultrasonic scalers and other technology for which maximum suction methods and rubber dam should be used as much as possible. Alternatively, procedure with minimal or no aerosol potential may be chosen, such as the approach of the Safe, Aerosol-free, Emergent (SAFE) Dentistry concept (28). New measures of aerosol control might be considered such as innovative suction technology, once their efficiency and evidence have been demonstrated.

The description of possible activities and risk mitigation measures under each control area is merely illustrative and not meant to be comprehensive, nor is the aim to provide specific guidance on actual measures that need to be taken in a specific setting or situation. Organizations and professional associations are encouraged to structure their guidance according to the five areas of control, thereby making it easier for clinicians to systematically follow and implement. Dental teams may also use the framework to review available information from different sources and to collate them to the five control areas, which simplifies staying updated and taking informed decisions about increasing, maintaining or loosening control measures.

Whatever the choices in the five control areas are, it is important to keep three fundamental principles in mind: consider every patient to be potentially infectious, focus on precautions to prevent droplet/aerosol transmission, and use the best possible PPE for protection of patients and staff (5). In contrast to OSHA's hierarchy of controls, all five control areas are equally important and need to be considered in order to provide the best possible occupational safety and infection control. The combination of measures in all five areas provides best possible safety and protection.

## The Continuum of Uncertainty and Risk Under COVID-19

Knowing the characteristics of a new infectious agent and its transmission mode, infectivity, disease patterns and many other aspects are part of the foundational determinants for an effective pandemic response, including appropriate infection control measures (29). Only when these aspects are known, the risks of certain events and outcomes can be fully assessed. This empirical analysis of observations, epidemiological surveillance, laboratory and virologic insights is highly complex and dynamic. It resembles a giant puzzle where over time different pieces are coming together toward a more complete picture. Less than a year after the first identification a lot is already known about SARS-Cov-2 and the clinical features of COVID-19 infections. Even though uncertainty is decreasing, and knowledge is increasing on a daily basis, reaching full understanding of the pandemic will take more time. Uncertainties about fundamental properties of the disease and the resulting risks will remain and impact on clinical decisions.

**Figure 2:**
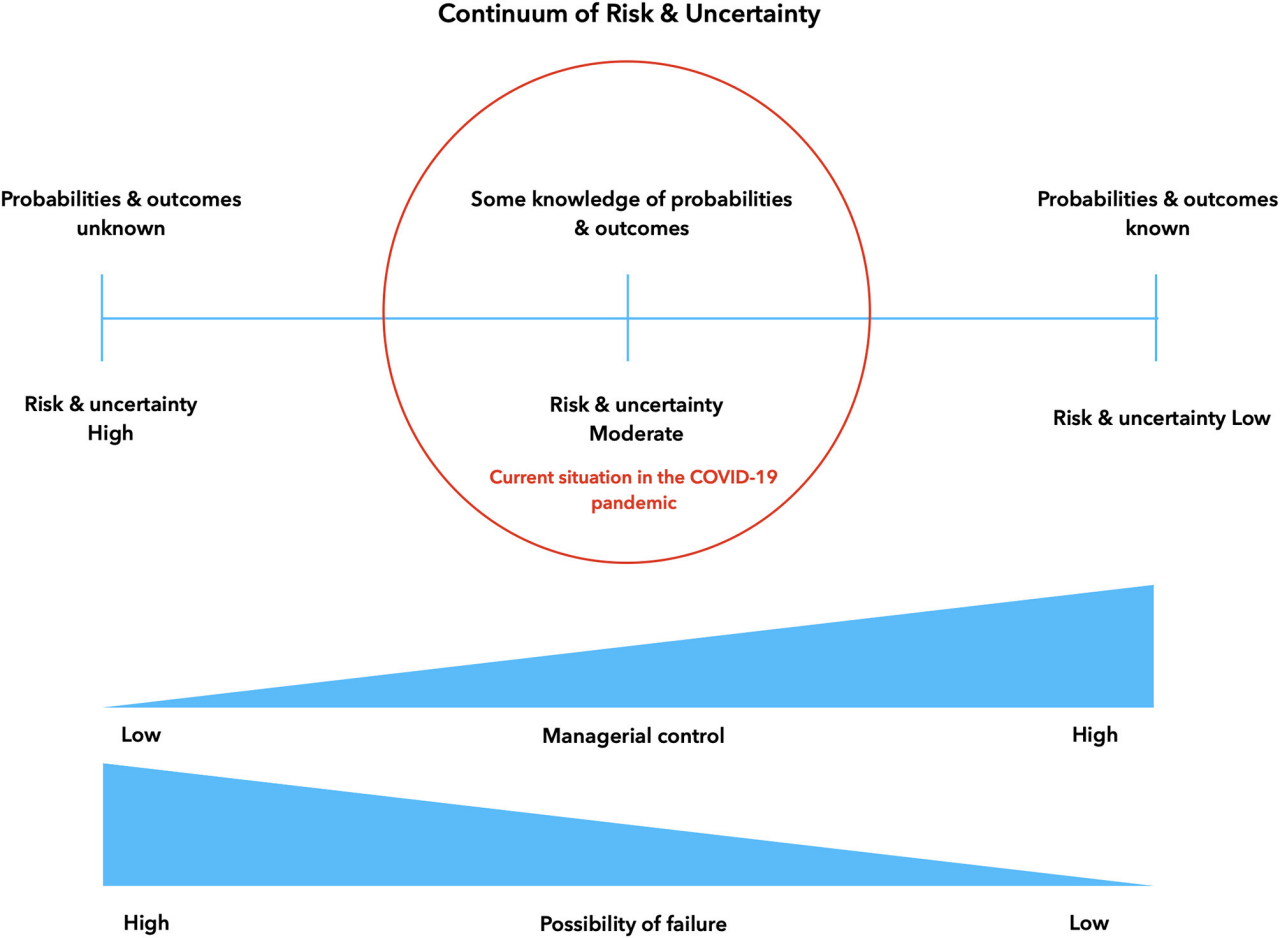
Continuum of risk, uncertainty and managerial control.

The role of airborne transmission *via* droplets and aerosols is one of the major areas of uncertainty for dentistry, since many of the frequently-used interventions are classified as aerosol-generating procedures (30, 31). Consequently, the current situation limits managerial control in dental settings and leaves a probability for failure (Figure 2). So far there have been no reports of major SARS-CoV-2 transmission events in dental settings, but several factors are limiting knowledge and reporting of such incidents; it is likely that more details will emerge over time (5, 32).

Understanding the dynamic nature of the situation, where progress toward full certainty and knowledge of risks may not be a linear process; and where new unexpected challenges may appear at any time, is important when deciding on adapting control and risk management in dental services to the COVID-19 pandemic. Ideally, such measures would need to be equally dynamic and adaptive in order to provide effective protection.

## Scenario Planning Using the Pandemic-5 Framework for COVID-19 Control in Dentistry

Systems thinking opens the door to a number of analysis and modeling approaches that help understand the relationships and interlinkages between different elements of the system (24). Scenario planning is among the tools that link problem-driven analysis with goal-oriented solutions (33). The principle of a “control knob” to step up or decrease precautions allows for flexible adaptation in the COVID-19 or other novel infectious agent contexts. External factors beyond control of the dental team, such as the level of community spread, availability of PPE or availability of a vaccine, are determining the adaptive measures within the control framework. The overall goal of the adaptations is to maintain and provide safe care for patients and clinicians, without the risk of infection or disease transmission.

## Shifting Responsibility for Pandemic Control and Response Requires High Compliance

When the first peak of the pandemic subsided, governments or states were allowing the gradual re-opening of dental services. This process implied shifting aspects of population-wide containment measures to clinic-level measures. By implication, individual practitioners and dental teams had to make the required clinical COVID-19 control decisions based on the most current guidance and their best professional judgment. This is a crucial step in devolving elements of the COVID-19 (or any future airborne infectious disease) preparedness and response, and one which requires confidence that appropriate measures will be uniformly followed.

However, dental teams express concerns over a multitude of uncertainties. These uncertainties include the understanding of community transmission, shortages of PPE, and safety of care without full PPE (34). On the other hand, previous studies show that dentists frequently tend to downplay risks and display a degree of over-confidence with their infection control measures, which may contribute to potentially lower compliance with new recommendations (35, 36). Others may even consider external guidance as an intrusion of their professional autonomy (37–39).

The Pandemic-5 Framework for COVID-19 Control in Dentistry provides an opportunity to simplify and systematize decisions from a clinical setting and practitioner perspective. The framework supports a comprehensive systems-driven approach by using dental clinics as a setting to integrate pandemic clinical responses with the implementation of appropriate infection control protocols. Traditionally these two aspects are addressed independently from each other in separate concepts.

The proposed framework shifts the locus of control back to the clinical setting and the provider's decision realm. Decisions are made based on rational assessments of uncertainty and risk, taking into account external uncontrollable factors. A clinician who feels in control, has decision autonomy and confidence, is more likely to adapt to change and to comply responsibly with service recommendations (40). The knowledge of the five control areas and options to fine-tune the pandemic response empowers and facilitates active management of the pandemic risk.

The concept also facilitates system adaptations in resource-poor settings where governmental or robust epidemiological guidance may not be available. Validation of the concept of the five pandemic control areas, using other international guidelines is encouraged and should not be too complex. Other areas of healthcare are facing similar challenges related to aerosols and proximity to patients, such as anesthesiology, maxillo-facial or ear-nose-throat surgery. Future research may show to what extent the Pandemic-5 Model might also be applicable to these clinical disciplines.

## Data Availability Statement

The original contributions generated for the study are included in the article/supplementary material, further inquiries can be directed to the corresponding author.

## Author Contributions

All authors contributed equally to conceptualizing, drafting, and finalizing the manuscript.

## Conflict of Interest

The authors declare that the research was conducted in the absence of any commercial or financial relationships that could be construed as a potential conflict of interest.
